# Diagnostic performance of plasma and urine neutrophil gelatinase-associated lipocalin, cystatin C, and creatinine for acute kidney injury in burn patients: A prospective cohort study

**DOI:** 10.1371/journal.pone.0199600

**Published:** 2018-06-26

**Authors:** Youngmin Kim, Yong Suk Cho, Dohern Kym, Jaechul Yoon, Haejun Yim, Jun Hur, Wook Chun

**Affiliations:** Department of Surgery and Critical Care, Burn Center, Hangang Sacred Heart Hospital, College of Medicine, Hallym University Medical Center, Seoul, Korea; The University of Manchester, UNITED KINGDOM

## Abstract

**Background:**

Diagnosing acute kidney injury quickly is imperative since it is known as an independent risk factor for mortality in burn patients. We evaluated the diagnostic power of creatinine, cystatin, serum and urine neutrophil gelatinase-associated lipocalin at different time periods and observed the changes from baseline for each biomarker.

**Methods:**

This was a prospective observation study from January 2015 to February 2016. A total of 84 patients were enrolled consecutively. Serum creatinine, serum cystatin C, and serum and urine neutrophil gelatinase-associated lipocalin were measured at admission, 7^th^, 14^th^, 21^st^, and 28^th^ days after admission. All samples were collected until acute kidney injury developed.

**Results:**

Acute kidney injury developed in 35 patients. The mean age was 49.6 years with a male predominance. The median urine neutrophil gelatinase-associated lipocalin was the lowest (11.6 ng/dL) at admission, and the highest at 85.5 ng/dL on day 7. Mean creatinine level was the highest (0.88 mg/dL) at admission and the median creatinine level was the lowest (0.56 mg/dL) on the 14^th^ day. The area under the curve of creatinine levels was the highest with 0.857 during the 1^st^ week. The area under the curve of urine neutrophil gelatinase-associated lipocalin was the highest with 0.803 during the 5^th^ week.

**Conclusions:**

Within 1 week of acute kidney injury, creatinine level was the optimal biomarker for diagnosis while urine neutrophil gelatinase-associated lipocalin showed better diagnostic performance following the 4- week period.

## Introduction

Severe burn injuries are one of the most devastating traumas with a high potential of mortality and morbidity. During burn treatment, acute kidney injury (AKI), known as an independent risk factor for mortality, is a frequent complication in patients with severe burns. In addition, it has been reported that burned patients with AKI have mortality rates ranging from 50% to 100%.[1] The mainstream treatment is prevention of the underlying insult, early diagnosis and rapid termination of nephrotoxic insults. Therefore, it is essential to identify early biomarkers that can predict AKI and to initiate intervention as soon as possible to prevent pathophysiologic events that lead to AKI. [1] However, serum creatinine, which is one of the criteria of AKI diagnosis and a reflection of renal function, is not an accurate biomarker because of the following clinical situations: a) serum creatinine levels do not increase until the glomerular filtration rate (GFR) decreases by 30–40%, b) serum creatinine levels can be affected by age, sex, weight, muscle mass, catabolic state, some medication, c) serum creatinine level can be lower in septic AKI because of reduced generation of creatinine and in critically ill patients because of fluid overload, which occurs frequently, d) serum creatinine can be increased in renal conditions such as prerenal azotemia which usually develops with dehydration and hypovolemic shock. These reasons limit the use of serum creatinine for early AKI detection and may result in missing of critical period for treatment of AKI; therefore, a quick and simple diagnostic tool is needed to detect a patient’s risk of AKI.[2] During the past few decades, serum cystatin C and plasma and urine neutrophil gelatinase-associated lipocalin (NGAL) levels have been introduced as early biomarkers for AKI as their levels were found to increase earlier than the levels of serum creatinine.[3] However, few studies have clarified the significance of associations between serum cystatin C, plasma NGAL, and urine NGAL levels and their ability to detect AKI in patients with major burn injuries. The purpose of our study was to observe the baseline changes of AKI biomarkers including serum creatinine, serum cystatin C, plasma NGAL and urine NGAL and to evaluate their diagnostic value in detecting AKI according to time changes in patients with severe burns.

## Materials and methods

### Selection and description of participants

From January of 2015 to February of 2016, we enrolled 84 patients for this prospective observational study. The inclusion criteria included the following: age >18 years and presence of burn wounds covering more than 20% of total body surface area (TBSA) requiring complete acute fluid resuscitation during the first 3 days of treatment. Our exclusion criteria were as follows: a) presence of a medical condition that influenced kidney function such as chronic kidney disease, liver disease, heart disease and diabetes mellitus b) having specific past medical history. We collected multiple data including clinical and laboratory findings of the enrolled patients through the electronic medical recording system of our hospital. In addition, we obtained written informed consent from all patients. If the patients were not able to give consent, consent was obtained from each patient’s spouse, a parent, or the child older than 18 years in order. Assessing patients on their ability to give consent was done by the physician and consent for participation in this study and for researchers to access a patient’s medical records associated with this study. This study was approved by the institutional review boards of Hangang Sacred Heart hospital (IRB N0.2015-086-2). Diagnosis of AKI was determined by the Acute Kidney Injury Network (AKIN) criteria. The AKIN criteria was used to identify kidney injury as follows: Stage 1: creatinine increased 1.5× baseline value or absolute increase in creatinine of at least 0.3 mg/dL within 48 hours or urine production of <0.5 mL/kg for 6 h, Stage 2: creatinine increased 2× baseline value or urine production of <0.5 mL/kg for 12 h, Stage 3: creatinine increased 3× baseline value or >4 mg/dL, absolute increase in creatinine within 48 h, urine output of <0.3 mL/kg for 24 h, or requiring continuous renal replacement therapy (CRRT), regardless of the status.[4] Because burns are an accidental event, there were no pre-admission creatinine values that could be used as a baseline for any of the patients. In patients with major burns, if creatinine value at admission is defined as a baseline measurement, this would result in an overestimation of true baseline creatinine levels because of hemoconcentration accompanied by burn shock. Therefore, the lowest value after burn shock resuscitation, within post-admission day 5, was chosen as the baseline creatinine value in each patient.[5]

### Technical information—Specimen collection and measurement of biomarkers

We collected blood and urine samples at admission and at the 7^th^, 14^th^, 21^st^ and 28^th^ day from admission until AKI was diagnosed in the AKI group. Once a patient was diagnosed with AKI, blood samples were no longer collected. All samples in the non-AKI group were collected weekly from admission until the 28^th^ day after admission. Plasma NGAL was measured using Triage NGAL reagent and Triage Meter (Alere Healthcare, San Diego, CA, SA). Serum cystatin C was measured by the turbidimetric immunoassay method using the HiSense cystatin C kit (HBi, Anyang, Korea) and Hitachi 7600 analyzer (Hitachi, Tokyo, Japan). Serum creatinine was measured by an enzymatic method using Cica Creatinine reagent (KANTO Chemical, Tokyo, Japan) and Hitachi 7600 analyzer (Hitachi, Tokyo, Japan). For urine NGAL, urine specimens were transferred to a centrifuge tube and centrifuged at ≥ 400 relative centrifugal force for a minimum of 5 minutes. The supernatant was then stored at -70°C for batch analysis. After thawing, specimens were mixed and centrifuged at 2500–3000 × g for 10 minutes prior to use to remove particulate matter and ensure consistency in the results. Urine NGAL was measured by chemiluminescence immunoassay using the Architect i2000SR analyzer (Abbott Diagnostics, Abbott Park, IL, USA) and dedicated urine NGAL reagent (Abbott Diagnostic, USA). All measurements were performed according to manufacturers’ instructions.

### Statistical analysis

All continuous variables distributed normally were expressed as means ± SD, and continuous variables distributed nonnormally as medians (interquartile rage [IQR]) and the frequencies of categorical variables were expressed as percentages. Continuous variables were analyzed with the independent *t*-test when there were normal distributions and with Mann-Whitney U-test when there was not a normal distribution. Categorical variables were assessed using the Chi-square test and Fisher’s exact test if appropriate. To compare the difference for each biomarker over the time, we used repeated measures ANOVA and a pairwise T test with Bonferrroni. The area under the curve (AUC) of the receiver operating characteristic (ROC) curve was used to evaluate the predictive accuracy of AKI for laboratory results and all values and outcomes of AKI patients up to each point of diagnosis of AKI were cumulatively included. The cut off value was calculated using the ROC curve with the Youden index. The AUC > 0.8 suggests excellent predictive power of diagnostic performance. P values < 0.05 were considered to be statistically significant. Statistical analyses were performed using R statistical software (https://cran.r-project.org).

## Results

### Patient characteristics according to the AKI development

Among 84 patients, AKI developed within 35 days in 35 patients and it did not develop within this time in 49 patients. The mean age was 49.6 years with male predominance. The percentage of TBSA burned was 46.2% and incidence rate of inhalation injury was 13.1%. Mean age, the percentage of TBSA burned, incidence rate of inhalation injury, and ABSI score were significantly higher in the AKI group. The usage rates of colistin, vasopressin, and CRRT were significantly higher in the AKI group. Overall mortality rate was 34.5% and significantly higher at 82.9% in the AKI group (Table 1). Among 35 patients who were diagnosed with AKI, 11 patients (AKIN1; 2 patients, AKIN2; 7 patients, AKIN3; 2 patients) were diagnosed by the 1^st^ week, 12 patients (AKIN1; 2 patients, AKIN2; 1 patient, AKIN3; 10 patients) during the 2^nd^ week, 2 patients (AKIN3; 2 patients, respectively) during the 3^rd^ and 4^th^ week and 8 patients (AKIN1; 2 patients, AKIN4; 7 patients, AKIN3; 2 patients) were newly diagnosed during the 5^th^ week.

**Table 1:** Demographics of the patients classified by the AKI development.

	Total patients (n = 84)	no AKI group (n = 49)	AKI group (n = 35)	p-value
Mean age	49.6±15.1	44.6±13.1	56.5±15.1	<0.001
Gender (male:female)	74:10	42:7	32:3	0.649
Mode (FB:SB:EB)	68:4:12	36:3:10	32:1:2	0.113
% TBSA burned	46.2±20.9	34.2±11.9	60.5±21.1	<0.001
% 3^rd^ burned area	20.0[10.0–42.5]	12.0[8.0–20.0]	49.0[28.5–65.0]	<0.001
Inhalation injury (%)	11 (13.1%)	1 (2.0%)	10 (28.6%)	<0.001
ABSI Score	9.0[7.0–11.0]	7.0[6.0–9.0]	11.0[9.0–12.5]	<0.001
APACHE II	14.5±10.2	8.7±5.7	22.8±9.3	<0.001
LOS	44.5[12.5–61.5]	51.0[44.0–66.0]	10.0[6.0–23.5]	<0.001
Supportive cares				
Colistin (%)	30 (35.7%)	8 (16.3%)	22 (62.9%)	<0.001
Vasopressor (%)	32 (38.1%)	1 (2.0%)	31 (88.6%)	<0.001
CRRT (%)	18 (21.4%)	0 (0.0%)	18 (51.4%)	<0.001
Death	29(34.5%)	0 (0.0%)	29 (82.9%)	<0.001

AKI, acute kidney injury; N, number; FB, flame burn; SB, scald burn; EB, electrical burn; TBSA, total body surface area; ABSI, Abbreviated Burn Severity Index; APACHE, acute physiology and chronic health evaluation score; LOS, length of hospital stay; CRRT, continuous renal replacement therapy

### The baseline changes of the biomarkers

**Fig 1:**
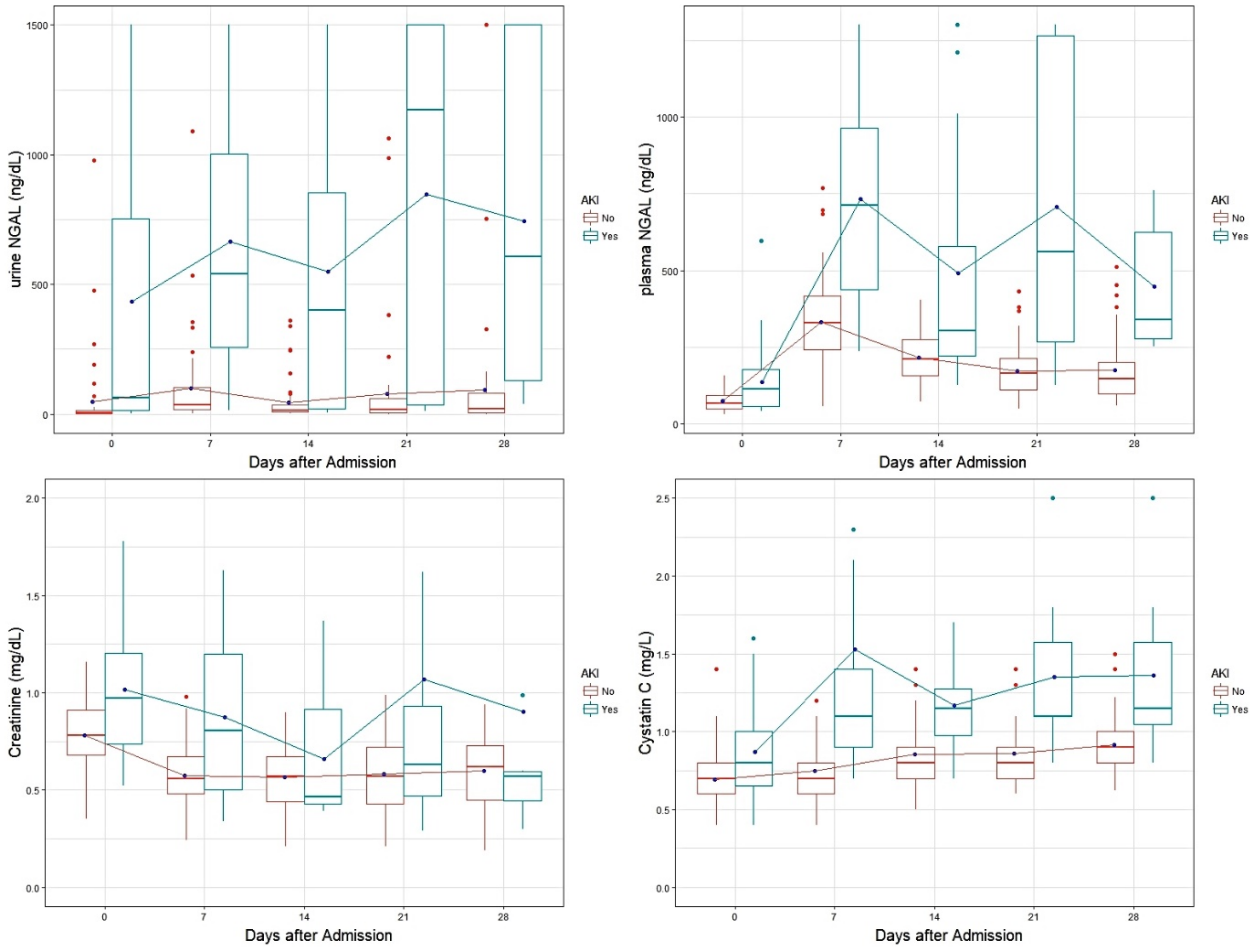
The changes in urine neutrophil gelatinase-associated lipocalin (NGAL) and plasma NGAL serum creatinine, and cystatin C levels between AKI and non-AKI groups at different time points.

First, we investigated the baseline changes of the biomarkers to find out unique features that are specific to severely burned patients. For all patients, the median urine NGAL was the lowest at 11.6 mg/dL at admission and the highest at 85.5 mg/dL on the 7^th^ day. The median plasma NGAL was the lowest at 74.5 mg/dL at admission and the highest at 464.6 mg/dL on the 7^th^ day. Mean creatinine level was the highest at 0.88 mg/dL at admission and the lowest at 0.56 mg/dL on the 14^th^ day. The median cystatin C level was the highest at 0.90 mg/dL on the 14^th^, 21^st^, and 28^th^ day and the lowest at 0.70 mg/dL at admission (Table 2). All biomarker levels were significantly lower in the non-AKI group, except the creatinine level, on the 14^th^, 21^st^, and 28^th^ days (Fig 1).

**Table 2:** Changes in biomarker levels over time between the AKI and non-AKI group.

Period	biomarkers	Total	Non-AKI	AKI	p-value
admission	Urine NGAL	11.6[3.9–83.2]	6.0[3.0–13.6]	62.8[13.9–754.6]	<0.001
	Plasma NGAL	74.5[53.0–127.0]	67.0[48.0–93.0]	113.0[57.5–176.5]	0.003
	Creatinine	0.88±0.30	0.78±0.20	1.02±0.36	0.001
	Cystatin C	0.70[0.60–0.80]	0.70[0.60–0.80]	0.80[0.65–1.00]	0.008
7^th^ day	Urine NGAL	85.5[21.1–353.9]	35.0[16.4–102.6]	540.7[238.7–1159.7]	<0.001
	Plasma NGAL	464.6±298.2	333.1±150.0	733.1±345.5	<0.001
	Creatinine	0.67±0.29	0.58±0.16	0.87±0.39	0.001
	Cystatin C	0.80[0.70–1.00]	0.70[0.60–0.80]	1.10[0.90–1.40]	<0.001
14^th^ day	Urine NGAL	18.9[8.0–60.2]	14.2[6.8–34.3]	401.1[16.8–1069.3]	0.002
	Plasma NGAL	228.0[159.0–282.0]	21.0[156.0–275.0]	302.5[207.5–722.0]	0.012
	Creatinine	0.56[0.43–0.67]	0.57[0.44–0.67]	0.46[0.42–0.92]	0.772
	Cystatin C	0.90[0.70–1.00]	0.80[0.70–0.90]	1.51[0.95–1.35]	0.001
21^st^ day	Urine NGAL	25.87.4–69.2]	18.5[5.5–61.0]	1173.2[28.6–1500.0]	0.001
	Plasma NGAL	172.0[119.0–245.5]	164.0[111.0–214.0]	561.0[241.0–1300.0]	<0.001
	Creatinine	0.57[0.43–0.75]	0.57[0.43–0.72]	0.72[0.47–1.13]	0.249
	Cystatin C	0.90[0.80–1.10]	0.80[0.70–0.90]	1.10[1.10–1.60]	<0.001
28^th^ day	Urine NGAL	39.9[6.5–112.5]	20.4[6.0–82.5]	607.4[99.6–1500.0]	0.001
	Plasma NGAL	165.5[102.0–251.0]	146.0[99.0–202.0]	339.0[276.5–664.0]	<0.001
	Creatinine	0.60[0.45–0.73]	0.63[0.45–0.73]	0.58[0.44–0.79]	0.800
	Cystatin C	0.90[0.80–1.10]	0.90[0.08–1.00]	1.15[1.00–1.65]	0.006

AKI, acute kidney injury; NGAL, neutrophil gelatinase-associated

### AUC for biomarkers and associated changes noticed each week once AKI was diagnosed

**Fig 2:**
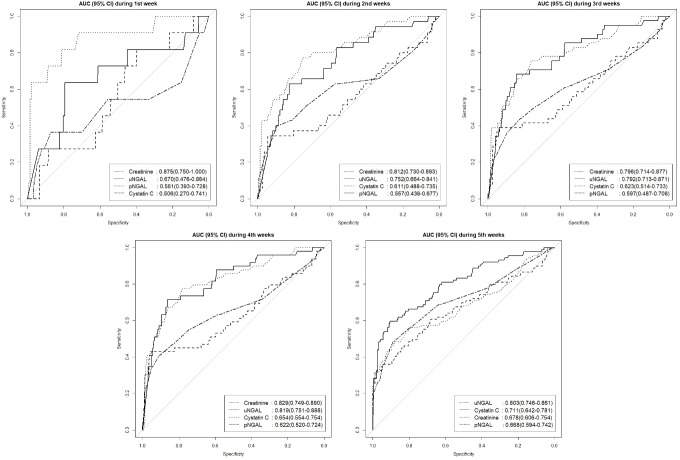
AUC for plasma NGAL, urine NGAL, creatinine, and cystatin C at weeks when AKI was diagnosed.

The period of AKI development was divided into a total of 5 weeks (from the 1^st^ week to 5^th^ week) to evaluate the diagnostic power for detection of AKI within each week. In addition, we also compare the 1^st^ and 2^nd^ place biomarkers to evaluate discrimination of each for the detection of AKI. Within the 1^st^ week, the AUC of creatinine was the highest with 0.875 and significantly higher than the 2^nd^ highest, urine NGAL, with 0.670 (p = 0.012). Within the 2^nd^ week, the AUC of creatinine was the highest at 0.812 and higher than the AUC of the 2^nd^ highest NGAL with 0.752; however, this was not significant (p = 0.256). Within the 3^rd^ and 4^th^ week, the AUC of urine NGAL was the highest (0.796 vs. 0.819, respectively) and the AUC of creatinine was the 2^nd^ highest at 0.792 and 0.819; however, this was not a significant difference (p = 0.935 vs. 0p = 0.995 respectively). Within the last week, the AUC of urine NGAL was the highest at 0.803 and significantly higher than the 2^nd^ highest AUC of cystatin C with 0.711 (p = 0.016) (Table 3). The AUC of creatinine declined from 0.875 to 0.680 over time, whereas AUC of urine NGAL, plasma NGAL and cystatin C increased from 0.670 to 0.803, from 0.561 to 0.668, and from 0.506 to 0.711, respectively. (Fig 2)

**Table 3:** Comparisons of the AUC between 1^st^ and 2^nd^ place of each biomarkers at each time period.

	1^st^ week	2^nd^ week	3^rd^ week	4^th^ week	5^th^ week
AUC at 1^st^	0.875 (creatinine)	0.812 (creatinine)	0.796 (creatinine)	0.819 (creatinine)	0.803 (uNGAL)
AUC at 2^nd^	0.670 (uNGAL)	0.752 uNGAL)	0.792 (uNGAL)	0.819 (uNGAL)	0.711 (Cystatine C)
P-value	0.012	0.256	0.953	0.995	0.016

AUC, area under the curve; uNGAL, urine neutrophil gelatinase-associated;

### Repeated biomarker assessments over the time period

Urine NGAL showed a significant difference according to group (F = 141.497, p < 0.001) and time (F = 1.968, p = 0.099) or interaction effect with group and time (F = 2.112, p = 0.079). Plasma NGAL showed a significant difference according to group (F = 123.28, p < 0.001) and time (F = 48.05, p < 0.001) or interaction effect with group and time (F = 13.96, p < 0.001). Creatinine showed a significant difference according to group (F = 56.615, p < 0.001) and time (F = 5.359, p < 001) or interaction effect with group and time (F = 1.707, p = 0.148). Cystatin C showed a significant difference according to group (F = 37.547, p < 0.001) and time (F = 5.195, p < 0.001) or interaction effect with group and time (F = 3.569, p = 0.007). Initial plasma NGAL and creatinine level showed different levels following 5 weeks by post hoc analysis with Bonferroni (Fig 1).

### Sensitivity and specificity of cutoff values for each biomarker (plasma and urine NGAL, creatinine and cystatin C)

We also calculated a cutoff value and test characteristic for each biomarker. The cutoff values of creatinine at admission was 0.87 mg/dL (sensitivity 0.818, specificity 0.811). From the 2^nd^ to the 4^th^ week, the cutoff value of creatinine was 0.78 mg/dL. The sensitivity and specificity during the 2^nd^ week were 0.771 and 0.753, respectively. The sensitivity and specificity were 0.756 and 0.761 respectively, during the 4^th^ week. During the 5^th^ week, the sensitivity and specificity were 0.776 and 0.780, respectively. The cutoff value of urine NGAL was 170.4 ng/dL with a sensitivity of 0.714 and specificity of 0.863 during the 4 th week. During the 5 th week, the urin NGAL was 159.2 ng/dL with a sensitivity of 0.596 and specificity of 0.906. Cystatin C showed the sensitivity with 0.483 and the specificity with 0.890 at a cutoff value of 1.10 mg/L during 5^th^ week. (Table 4)

**Table 4:** Cutoff value and sensitivity and specificity at the time of AKI diagnosis.

Period	biomarkers	Cut-off value	Sensitivity(95% CI)	Specificity(95% CI)	AUC(95% CI)
1^st^ week	Urine NGAL	170.4	0.636(0.308–0.891)	0.793(0.744–0.835)	0.670(0.476–0.864)
	Plasma NGAL	233.0	0.396(0.343–0.452)	0.818(0.482–0.977)	0.561(0.393–0.728)
	Creatinine	0.87	0.818(0.482–0.977)	0.811(0.764–0.852)	0.875(0.750–1.000)
	Cystatin C	1.20	0.364 (0.109–0.692)	0.870(0.828–0.905)	0.506(0.270–0.741)
2^nd^ week	Urine NGAL	170.4	0.714(0.537–0.854)	0.667(0.609–0.721)	0.752(0.664–0.841)
	Plasma NGAL	595.0	0.343(0.191–0.522)	0.943(0.911–0.967)	0.557(0.438–0.677)
	Creatinine	0.78	0.771(0.599–0.896)	0.753(0.700–0.800)	0.812(0.731–0.893)
	Cystatin C	1.20	0.400(0.239–0.579)	0.893(0.852–0.926)	0.611(0.487–0.735)
3^rd^ week	Urine NGAL	170.4	0.683(0.519–0.819)	0.843(0.796–0.883)	0.792(0.713–0.870)
	Plasma NGAL	493.0	0.390(0.242–0.555)	0.935(0.901–0.961)	0.597(0.487–0.707)
	Creatinine	0.78	0.756(0.597–0.876)	0.761(0.708–0.809)	0.796(0.714–0.877)
	Cystatin C	1.10	0.439(0.285–0.603)	0.823(0.774–0.865)	0.623(0.514–0.733)
4^th^ week	Urine NGAL	170.4	0.714(0.567–0.834)	0.863(0.818–0.901)	0.819(0.750–0.888)
	Plasma NGAL	493.0	0.429(0.288–0.578)	0.951(0.919–0.973)	0.622(0.520–0.724)
	Creatinine	0.78	0.776(0.634–0.882)	0.780(0.726–0.826)	0.819(0.749–0.890)
	Cystatin C	1.20	0.408(0.270–0.558)	0.909(0.869–0.940)	0.654(0.554–0.754)
5^th^ week	Urine NGAL	159.2	0.596(0.486–0.698)	0.906(0.862–0.940)	0.803(0.746–0.860)
	Plasma NGAL	431.0	0.360(0.261–0.468)	0.947(0.911–0.971)	0.668(0.594–0.742)
	Creatinine	0.87	0.472(0.365–0.581)	0.886(0.839–0.923)	0.680(0.606–0.754)
	Cystatin C	1.10	0.483(0.376–0.592)	0.890(0.844–0.926)	0.711(0.641–0.781)

AKI, acute kidney injury; NGAL, neutrophil gelatinase-associated; AUC, area under the curve; CI, confidence interval

## Discussions

AKI is a crucial health problem in critically ill patients including those with burns. Early detection of AKI and subsequent adequate treatment could improve patient outcomes. However, AKI is determined mainly by creatinine and urine output which are insensitive and nonspecific diagnostic factors that reflect renal function. Ideal biological markers are required for the early diagnosis of AKI with good sensitivity and specificity. We evaluated the diagnostic power of plasma and urine NGAL, cystatin C, and creatinine levels in the post-burn period for predicting AKI in patients with major burn injuries by the time AKI developed.

Cystatin C is a representative marker of kidney function and not influenced by muscle, age, gender and protein intake unlike creatinine [6]. It is a 133-kDa cysteine proteinase inhibitor protein, which has half the life of creatinine (90–120 min compared to 4 h), so the serum level of Cystatin C changes earlier than that of creatinine.[7, 8] Many studies have shown that the performance of serum Cystatin C for the detection of AKI is superior to that of serum creatinine in various clinical settings.[8, 9] Yim et al. showed that serum Cystatin C is a more valuable diagnostic marker of AKI in major burn patients than serum creatinine.[5] However, Cystatin C showed a relatively lower AUC (the highest AUC was 0.711 during 5^th^ week) and creatinine showed a higher AUC throughout the study period except during the 5^th^ week in this study. It was inferred that this result was because AKI was diagnosed using creatinine and cystatin C levels which can be affected by factors other than renal filtration, such as systemic inflammation, which is frequently observed in burned patients with a high risk of infection. [10]

Neutrophil gelatinase-associated lipocalin (NGAL) has also emerged as a promising non-invasive biomarker of kidney injury.[11] Neutrophil gelatinase—associated lipocalin (NGAL) is a molecule that is released by neutrophils and epithelial cells, including the kidneys, trachea, lungs, and intestine. NGAL secreted by the glomerulus is reabsorbed by the proximal tubules. When there are renal insults, NGAL rapidly accumulates in the plasma because of a decrease in the glomerular filtration rate and in the urine because of reduced reabsorption and elevated release by the nephrons, and the accumulation precedes an increase in serum creatinine [12, 13] Recent studies reported that urine and plasma NGAL levels can increase rapidly after tubular injury and reflects severity of renal injury.[14, 15] In this study, urine NGAL showed a higher AUC than plasma NGAL during the study period.(Fig 2) The highest AUC in plasma NGAL was 0.668 during 5^th^ weeks, whereas urine NGAL showed AUC over 0.7 except during the 1^st^ week (0.752 during the 2^nd^, 0792 during the 3^rd^, 0.819 during the 4^th^ and 0.803 during the 5^th^ week). These differences between urine and plasma NGAL suggest that plasma NGAL may be influenced more easily by the circulating plasm volume changeable according to the condition of the patient with the burn (ie those with SIRS, sepsis and ARDS than urine NGAL.)

Creatinine is known to be a less sensitive biomarker affected by other causes. However, creatinine showed a better diagnostic performance than urine NGAL during the 1^st^ week (0.875 vs 0.670, p = 0.012). It is possible that the causes of AKI in patients with burn are different and classified according to the time period of AKI development.[16] Early AKI is associated with the volume given under fluid resuscitations and the degree of burn shock, whereas late AKI is caused by sepsis, nephrotoxic agents and multiorgan failure.[17, 18] Therefore, in the early stages, serum creatinine seems to reflect indirectly the volume deficiency due to burn shock rather than the renal function due to renal injury. Therefore, biomarkers should be selected according to the time course and causes of AKI.

The design of many studies evaluating biomarkers for various clinical settings in critically ill patients calls for laboratory data to be obtained at the earliest point of ICU admission. However, major patient with major burns go through a unique clinical course, such as non-septic sustained SIRS and relapse sepsis, until their wounds are healed. Inflammation can affect the levels of biochemical markers.[5] This study was designed in a way that laboratory data were obtained at admission, 7^th^, 14^th^, 21^st^ and 28^th^ day from admission. This method is optimal for detecting the trends of markers and may help to better interpret the biomarker.

However, this study has some limitations. First, there was no pre-admission baseline creatinine level for any of the patients included in this study. We set the baseline creatinine as the lowest level in the first 5 days. Therefore, this could potentially cause a misdiagnosis; however, there were no patients who were misdiagnosed with AKI due to hemoconcentration caused by the burn. Second, this study was conducted on a small group and performed in a single-center design. However, our burn center is the only burn facility operated by the University, and was designated as “The Emergency Center for Burn Care” by the Ministry for Heath, Welfare, and Family Affairs in Korea. Most patients who experience major burns are transferred here from across the nation. Therefore, although this was a single-center study, our patient sample was representative of Korean patients with burns. Third, disparity in the %TBSA and age exists between the AKI group and the non-AKI group. That is why %TBSA and age are well acknowledged powerful factors that are related to many complications and clinical outcomes in major burn patients.[5, 9, 19] Thus, it was difficult to evaluate the risk factors for mortality according to whether AKI was present or not. Fourth, we cannot ascertain whether the cut-off value for predicting AKI was obtained optimally because blood sampling was performed on weekly basis. Notwithstanding this limitation, the strengths of our study include our design of a prospective observational study which showed the baseline changes of the AKI biomarkers including plasma NGAL, urine NGAL, serum cystatin C, and serum creatinine and the diagnostic value of the biomarkers for detection of AKI during each week in the patients with severe burns. In our next study we plan to examine the clinical outcomes of patients and their association with various injury markers for AKI (e.g. interleukin-18, fatty acid binding proteins and kidney injury molecule 1) which are showing promise in the management of patients with this condition.

## Conclusions

In early AKI during the 1^st^ week, the creatinine level showed better diagnostic performance and urine NGAL showed better diagnostic performance in late AKI during the 5^th^ week. During the 2^nd^ to 4^th^ weeks, creatinine and urine NGAL showed similar diagnostic performance. This is because the causes of early and late AKI in burn patients are different and the baseline course of biomarker level over time are also different. Therefore, the time and baseline level of biomarker should be considered when the biomarkers are interpreted.

## Supporting information

S1 Dataset Datasets of this study. (CSV)
